# Knowledge Gain and the Impact of Stress in a Fully Immersive Virtual Reality–Based Medical Emergencies Training With Automated Feedback: Randomized Controlled Trial

**DOI:** 10.2196/67412

**Published:** 2025-06-04

**Authors:** Marco Lindner, Tobias Leutritz, Joy Backhaus, Sarah König, Tobias Mühling

**Affiliations:** 1 Institute of Medical Teaching and Medical Education Research, University Hospital Würzburg, Joseph-Schneider-Strasse 2, Würzburg, 97070, Germany, 49 931-201-70627

**Keywords:** virtual reality, VR, emergency medicine, knowledge gain, electrodermal activity, skin conductance, stress, emergency, medical emergencies, randomized controlled trial, simulation training, simulation, feedback, automated feedback, medical education, virtual patient

## Abstract

**Background:**

A significant gap exists in the knowledge and procedural skills of medical graduates when it comes to managing emergencies. In response, highly immersive virtual reality (VR)–based learning environments have been developed to train clinical competencies. However, robust evidence on how VR-based methods affect both short- and long-term learning outcomes, as well as physiological and perceived stress, remains limited.

**Objective:**

This study aimed to assess the effectiveness of VR-based simulation training, augmented with automated feedback, compared with video seminars at improving emergency medical competency among medical students. Furthermore, the study investigated the relationship between learning outcomes and physiological stress markers. The evaluation of participants’ perceived stress and estimated learning success was also performed to provide a more comprehensive insight into VR’s potential role in emergency training.

**Methods:**

In total, 72 senior medical students underwent VR-based emergency training (intervention) or viewed video seminars (control) on 2 topics (acute myocardial infarction and exacerbated chronic obstructive pulmonary disease) in an intraindividual crossover design. Levels of applied knowledge were assessed objectively by open-response tests pre- and postintervention and after 30 days. In addition, 2 electrodermal activity markers representing physiological stress response were measured during VR sessions using a wearable sensor. Participants also rated their estimated learning success and perceived stress. They also completed self-ratings of perceived stress and estimated learning success.

**Results:**

Short-term knowledge gains were comparable between the VR (mean 26.6%, SD 15.3%) and control (mean 27.2%, SD 16%) condition. However, VR training produced significantly higher long-term knowledge gains (VR: mean 17.8%, SD 15.1% vs control: mean 11.9%, SD 18%; difference: –5.9, 95% CI –11.5 to –0.4). Overall retention scores were likewise higher for VR (mean 75.4%, SD 12.5%) than for video-based learning (mean 69.0%, SD 14.5%), a difference that was more pronounced in the myocardial infarction scenario. Participants rated the VR format as significantly more effective (mean 4.83, SD 0.41, on a 5-point scale) than the video seminar (mean 3.44, SD 1.00). While physiological stress markers increased during VR sessions, their correlation with knowledge gains was weak and negative. No significant relationship was detectable between perceived stress and objective learning outcomes.

**Conclusion:**

VR-based simulation training with automated feedback may offer long-term learning advantages over a traditional video seminar in emergency-medicine education. Given the time constraints and resource limitations of clinical education, self-moderated VR-based learning may represent a valuable addition to conventional training methods. Future research could investigate the learning effects of VR scenarios regarding the retention of practical skills, as well as the impact of repeated or team-based scenarios.

## Introduction

Although emergency medicine constitutes a fundamental component of most medical education curricula, existing evidence highlights deficiencies in differential diagnosis, initial treatment, and verbal handover during medical emergencies among graduates [1-3]. In response to such deficits, highly immersive virtual reality (VR) learning environments have been developed to train resuscitation [4], clinical reasoning [5], team communication among paramedics [6], and the use of specific medical equipment [7]. A significant advantage of these software-based simulations is the potential incorporation of automated feedback (eg, insights into beneficial or detrimental actions and end-of-session evaluations), which can enhance learning even in the absence of a tutor. The acceptance and educational achievement of VR-based training programs are generally rated highly by participants [8-10], fostering the belief that they will play a crucial role in the future of emergency training. So far, most studies focus on subjective parameters such as usability, satisfaction, and self-reported learning success [5,6,11-13]. However, self-reported learning outcomes are criticized as being prone to errors [14,15] and in the context of VR-based simulations in emergency medicine, objective learning outcomes have not been systematically investigated to meet the quality criteria of meta-analyses [9,16].

When evaluating the learning outcomes of VR-based training, it is important to consider that immersive environments may influence the learning process in various ways. Evidence strongly supports the notion that VR increases learner motivation and improves the contextualization of the learning experience [17]. In a recent research project of ours, we explored different types of perceived stress encountered during VR-based training in medical emergencies [13]. We identified two key stressors within the training scenario, exerting influence on participants’ estimated learning success: (1) the control over the clinical situation and (2) the challenge of making decisions based on knowledge and skills. The first exhibited an inverse U-shaped relationship with estimated learning success, meaning that moderate control was beneficial, while too little or too much reduced estimated learning success. For the second, a higher perceived demand for knowledge and skills showed a negative correlation with estimated learning success. These observations align with those of similar studies, in which excessive stress was found to undermine cognitive abilities [18-20].

Stress, undeniably, encompasses both psychological and physiological components. A more comprehensive understanding of stress responses could thus be achieved through the investigation of perceived stress along with physiological stress markers [21]. Electrodermal activity (EDA) as a measure of sympathetic nervous system activity is frequently used to monitor psychophysiological activation in stressful conditions continuously [22]. EDA includes various parameters, among which the skin conductance level (SCL) and the frequency of nonspecific skin conductance responses (nsSCR) are particularly suitable for assessing background psychophysiological activation in the absence of specific stimuli [23]. Both physiological stress markers can be recorded by wearable sensors (eg, wristbands), causing minimal or no disruption to the simulation [24,25].

To evaluate objectively the learning outcomes of self-moderated VR-based emergency training with automated feedback and simultaneously to gain insight into the impact of different stress dimensions on the learning process, we aimed to answer the following questions. First, whether VR-based emergency simulation training is superior to video seminars for short- and long-term learning outcomes. Second, how perceived stress and physiological stress markers correlate during VR training sessions. Finally, to what extent perceived stress, physiological stress markers, and learning outcomes differ across various demographic groups and experience levels.

## Methods

### Fully Immersive VR-Based Simulation Training and Automated Feedback

STEP-VR (Simulation-based Training of Medical Emergencies for Physicians Using Virtual Reality; version 0.11b; ThreeDee GmbH, Munich, Germany) was used as the VR simulation of complex emergencies, which represents a joint development between a 3D visualization company and the University Hospital of Würzburg. The previously described hardware setup [13] comprised a Schenker XMG Core 15 Laptop (Schenker Technologies GmbH; chipset: Intel Core i7-9750H, 6×2.6 GHz; graphics adapter: Nvidia GeForce GTX 1650, 4 GB GDDR6 VRAM) and an Oculus Rift S (Lenovo Technologies and Oculus VR) VR head-mounted display.

For the intervention, 2 VR-based case scenarios from the subject of internal emergency medicine were used: (1) chest pain resulting from acute myocardial infarction and (2) dyspnea caused by exacerbation of chronic obstructive pulmonary disease. Both scenarios were developed by board-certified specialists in internal medicine and emergency medicine with additional qualifications in medical education, based on guidelines from the relevant professional societies [26,27]. The medical content of the scenarios and their evaluation have been described in detail in studies by Rickenbacher-Frey et al [12] and Mühling et al [13].

Furthermore, for this study, the software was complemented with an automated feedback system, providing recommendations based on medical guidelines. This system included various feedback modalities:

1. Positive notifications for correctly executed actions.
2. Warnings for actions that could potentially be harmful.
3. Prompts for missed time-critical actions using a “scaffolding” approach, in which additional cues are provided to guide the user without directly specifying the required action.
4. Access to the results of diagnostics (eg, computed tomography scans) through a virtual representation of a patient documentation system.
5. A final evaluation presented in a checklist format, indicating the completion of relevant actions.

**Figure 1.**
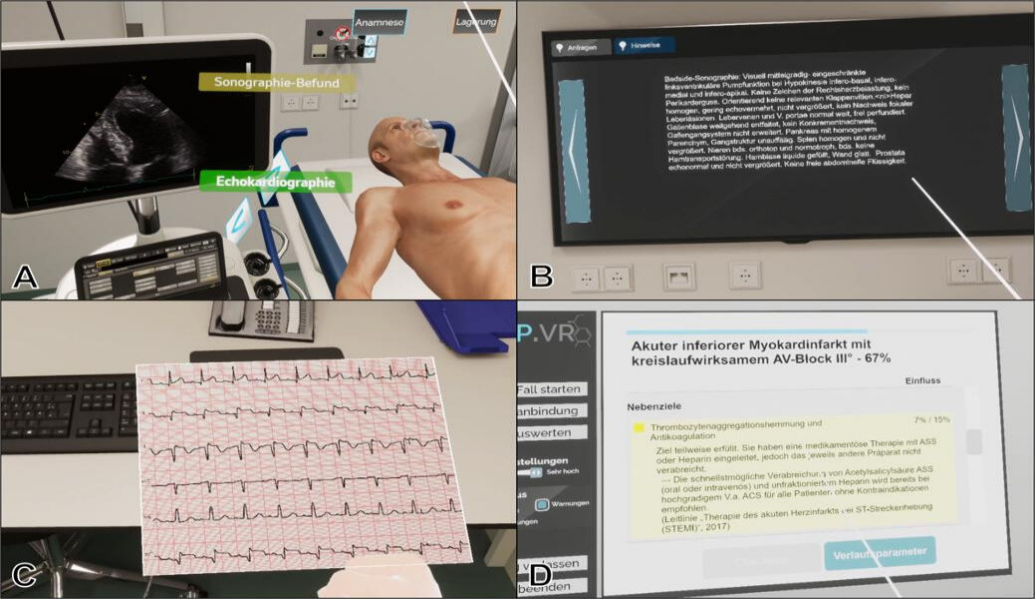
Feedback components in the adapted software. (**A**) Positive notifications (green) for correctly executed actions, (**B**) results of diagnostics in the virtual computer menu or (**C**) as direct output from medical devices (parameters calculated by dynamic physiology), and (**D**) final evaluation in checklist format.

For points 1‐4, brief messages, accompanied by an acoustic signal, were displayed immediately following the action. Users also had the option of accessing more detailed feedback through the virtual computer menu. Feedback components in the adapted version of STEP-VR are illustrated in Figure 1 and in real time during the use of the software are illustrated in Multimedia Appendix 1.

### Study Design and Data Collection

The study was conducted at the skills lab of a German medical school with a 6-year medicine degree curriculum between June 2022 and May 2023. In order to align with the study design standards of recent meta-analyses, we adopted a randomized controlled design. To ensure a uniform baseline knowledge level, only medical students who had passed the written examination in internal medicine (regularly at the end of the eighth semester) were eligible to enroll. In addition, students with known epilepsy or severe simulation sickness were not considered eligible for this study. Recruitment was through semester mailing lists, with participants being offered a €15 (approximately US $16.98) voucher as an incentive. The simulation training itself took place outside regular teaching hours in the form of individual sessions. To avoid motivation bias among participants in a potential control group, we used an intraindividual cross-over design. This design required all participants to complete 2 scenarios, each lasting 35 minutes—one using VR (intervention condition) and the other in the form of a video seminar (control condition).

At the start of the intervention condition, all participants completed a 5-minute VR tutorial, during which they could familiarize themselves with the functionality of the empty virtual emergency room while listening to explanations via an audio commentary. Following the tutorial, participants engaged in 20 minutes of a self-directed learning activity within the scenario, during which automated feedback was provided (learning phase [LP]). During this self-moderated period, users decided which measures they wanted to perform. Afterward, participants reviewed the final evaluation checklist in the virtual doctor’s office. To enhance content retention, they were given an additional 10 minutes to revisit the scenario and practice the medical procedures correctly (repetition phase [RP]). Throughout the whole session, a student tutor was available for technical support. As a control condition, video seminars for scenarios A and B lasting 35 minutes each were created comprising the same learning content in the form of a slide-based presentation. The lecturer interspersed content delivery with questions designed to foster reflection among the learners. Similar to the intervention, during the last 10 minutes participants were free to memorize the final summary or review specific slides from the presentation. All participants were randomly assigned to one of 2 groups. The first started with case A as VR scenario and then continued with case B as video seminar, while the second started with case A as video seminar and continued with case B as VR scenario. The random allocation sequence of whether case A or B was completed as an intervention or control condition was conducted by ML using computer-generated random numbers, who assigned participants to the intervention. Blocks of size 4 were used, ensuring that the groups remained comparable throughout the recruitment process.

**Figure 2.**
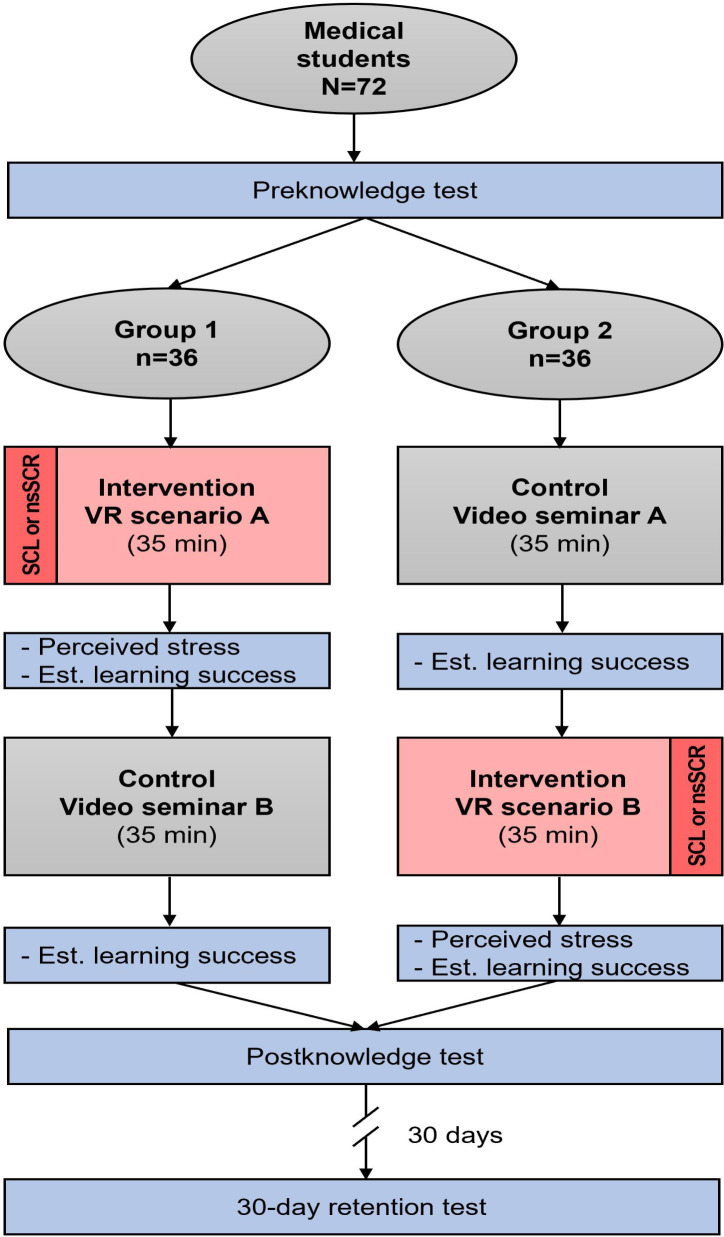
Study design and data collection process for knowledge tests and estimated learning success, as well as physiological stress markers (skin conductance level and nonspecific skin conductance response) and perceived stress during intervention and control. Participants completed one scenario in VR and another as a video seminar. Est: estimated; nsSCR: nonspecific skin conductance response; SCL: skin conductance level; VR: virtual reality.

Students were asked to take applied knowledge tests with open-ended questions on the 2 case scenarios, both before and after the intervention or control session, following a pre- and posttest design. In addition, students completed a web-based survey, in which they rated their estimated learning success (after both the intervention and control) and perceived stress (only following the intervention), along with providing person-specific data. To assess knowledge retention, a retention test took place in a separate in-person session 30 days later under the supervision of the raters. Figure 2 provides an overview of the study design and data collection process.

Assuming a moderate effect size, a significance level of *P*=.05, and a power of 80%, the estimated sample size was approximately 63 participants. To account for a projected dropout rate of 10% before retention, we adjusted the total number of participants to 72.

### Questionnaires and Measures

#### Knowledge Tests

To assess the objective learning outcome, 8 open-ended questions were created based on the scenario content, adhering to the guidelines provided by professional societies [26,27]. These questions focused on applied knowledge related to diagnosing and initiating treatment in the 2 emergency scenarios. Questions covered topics that were taught in both the intervention and control conditions. A corresponding rubric was developed (Multimedia Appendix 2) and tested during a pilot phase with a small number (n=8) of participants. It was subsequently refined to achieve optimal discriminative power. Participants could score a maximum of 1‐3 points for correct answers, depending on the difficulty and complexity of the content. Student performance was rated according to the percentage of correctly answered questions. The pre-, post-, and 30-day retention tests of knowledge all featured identical open-ended questions. Correct answers were never disclosed to participants. The responses were evaluated by 2 independent reviewers (a board-certified specialist in internal medicine with additional specialization in medical education [TM], and a medical student and trained skills laboratory tutor with 3 years of experience in rating of Objective Structured Clinical Examinations [ML]) in a blinded manner. The raters were unaware of which training modality (intervention or control) was delivered and at which time point (pre, post, or retention) the knowledge test was performed. In cases of disagreement between the raters, the respective answers were considered correct.

Short- and long-term knowledge gains were calculated as the differences between the posttest or 30-day retention test and the pretest, respectively.

#### Self-Assessment Questionnaires

##### Perceived Stress

In a previous study [13], we identified 3 main stress factors in VR-based emergency training: “sense of control” (ability to impact patient outcomes), “challenge to perform” (difficulty in making correct decisions using specific knowledge), and “social interaction to learn” (feeling of being watched and judged by others). Owing to time constraints and practical considerations, this study assessed these factors with just 1 relevant item each, based on their strong performance in the previous exploratory factor analysis. Students rated items on a 5-point Likert scale with 1 representing the minimum and 5 the maximum value. A separate overall rating for “total perceived stress” was evaluated based on a single item using the same scale. It should be noted that the questionnaire was used exclusively for the intervention (VR).

For the correlation analysis, the average of all 4 items (3 factors+total perceived stress) was calculated and labeled as “average perceived stress.”

##### Estimated Learning Success

A similar approach was taken for the student self-assessment of estimated learning success represented by 2 factors previously identified as “didactic value” (participants’ perceived general effectiveness of VR simulation) and “individual learning benefit” (the positive impact on the theoretical or practical skills of each participant). In this study, each factor was also evaluated with only 1 single item on the described 5-point Likert scale. Survey questionnaires with their items are listed in Multimedia Appendix 3. This questionnaire was used for both the intervention and the control condition.

To calculate the correlations, the average of the 2 items was taken and labeled as “average estimated learning.”

### Demographic Information

Demographic data were collected following the questionnaires on perceived stress and estimated learning success. It included questions on gender, academic semester, and previous experience in emergency medicine (eg, voluntary service and clinical rotation). In addition, participants were asked about the frequency of 3D-application use and their cumulative experience with VR applications.

### Measurement of Physiological Stress Markers

EDA was measured using the Empatica E4 wristband, following the manufacturer’s guide and good practice recommendations [23]. As there were no defined stimuli present during the VR scenarios, we opted to measure the continuous parameters SCL and nsSCR, suited to capture spontaneous fluctuations in EDA in the presence of an ongoing sustained stimulus over a period of time [28]. During completion of the tutorial, 3 minutes of baseline data were recorded for each participant. Recording during the intervention phase was divided according to the study design into the LP and RP. SCL and temperatures of all recordings were within normal ranges of 0.05-60 µs and 30‐40°C, respectively [29]. Data were processed with R (Version 4.3.1, RRID:SCR_001905; R Core Team) using the wearables package [30]. We calculated the median values of SCL and nsSCR per minute during the respective observation phases (baseline, LP, and RP), resulting in 6 parameters.

For correlation analysis, we wanted to account for the interindividual variability of SCL and nsSCR, as well as for the level of stress generated using the VR environment itself (without emergency scenario). Thus, the values of the individual baseline recording were subtracted from LP and RP data for every participant, yielding 4 parameters (2 baseline-corrected physiological stress markers combined with 2 observation phases): SCL (LP), SCL (RP), nsSCR (LP), and nsSCR (RP).

### Statistical Analysis

All data were analyzed by using descriptive statistics such as counts (both absolute and percentages), means, and SDs. Regarding primary outcome data from knowledge tests, differences and 95% CIs were also reported. As most of the parameters were not distributed normally (eg, knowledge tests and measures of EDA), repeated measures ANOVA was not an option. Likewise, the Friedman test was not suitable for a 2×3 design (2 groups at 3 time points). Therefore, the Wilcoxon rank-sum test was used for comparisons between the intervention and control conditions. Spearman rank test was used to calculate correlation coefficients (ρ). Curvilinear relationships were calculated using quadratic regression analyses, and the fit of the models was expressed by *R*². The interrater reliability was calculated using Cohen κ to quantify the agreement between the assessments of the independent reviewers. For all calculations excluding EDA data, GraphPad Prism (version 10.1.2, RRID:SCR_002798; GraphPad Software Inc) was used. Reporting was primarily conducted in accordance with the CONSORT-EHEALTH (Consolidated Standards of Reporting Trials of Electronic and Mobile Health Applications and Online Telehealth) recommendations [31]. The corresponding checklist is provided in Checklist 1.

### Ethical Considerations

#### Human Participant Ethics Review Approval

The local institutional review and ethics board judged the project as not representing medical or epidemiological research on human participants and as such adopted a simplified assessment protocol. The project was approved without any reservation under the proposal 20210917‐01.

#### Trial Registration

This study was not registered as a clinical trial as it examined an educational intervention (VR-based training) rather than a clinical or therapeutic measure. The primary outcomes focused on knowledge acquisition and learning effectiveness rather than patient-related health outcomes.

#### Informed Consent

Students were informed about the study, and their participation was voluntary. Written informed consent was obtained in printed form from all participants, who were also provided with information on data processing for the analysis and the publication of results. Contact details were supplied for participants wishing to withdraw their consent to data processing. The decision to participate or not had no consequences on the student’s academic progress.

#### Privacy and Confidentiality

Survey data from the questionnaires were collected anonymously using the EvaSys platform (Evasys GmbH). Data were processed and stored in accordance with local data protection laws.

#### Compensation Details

A €15 (approximately US $16.98) book voucher was handed to the participants upon completion of the retention test.

## Results

### Participants, Knowledge Tests, and Estimated Learning Success

A total of 72 participants were recruited for the study. All participants also completed the 30-day retention test, resulting in no dropouts. Person-specific factors are listed in Table 1. The gender distribution (46/72, 64% female participants) was representative of students in the degree program in medicine. Overall, there was only little previous experience with VR and 3D applications.

**Table 1.** Person-specific factors of participants.

Characteristics and experiences and subgroup		Value (N=72), n (%)	
Gender			
Female		46 (64)	
Male		25 (35)	
Diverse		1 (1)	
Academic semester			
8		20 (28)	
9		23 (32)	
10		26 (36)	
˃10		3 (4)	
Experience in emergency medicine (eg, voluntary service and clinical rotation)			
Yes		32 (44)	
No		40 (56)	
Frequency of 3D-application use			
Never		49 (68)	
Rarely		12 (17)	
Sometimes		3 (4)	
Frequent		7 (10)	
Daily		1 (1)	
Cumulative experience with VR^a^-applications			
None		30 (42)	
<1 h		26 (36)	
1‐5 h		13 (18)	
6‐10 h		1 (1)	
>10 h		2 (3)	

a VR: virtual reality.

**Figure 3.**
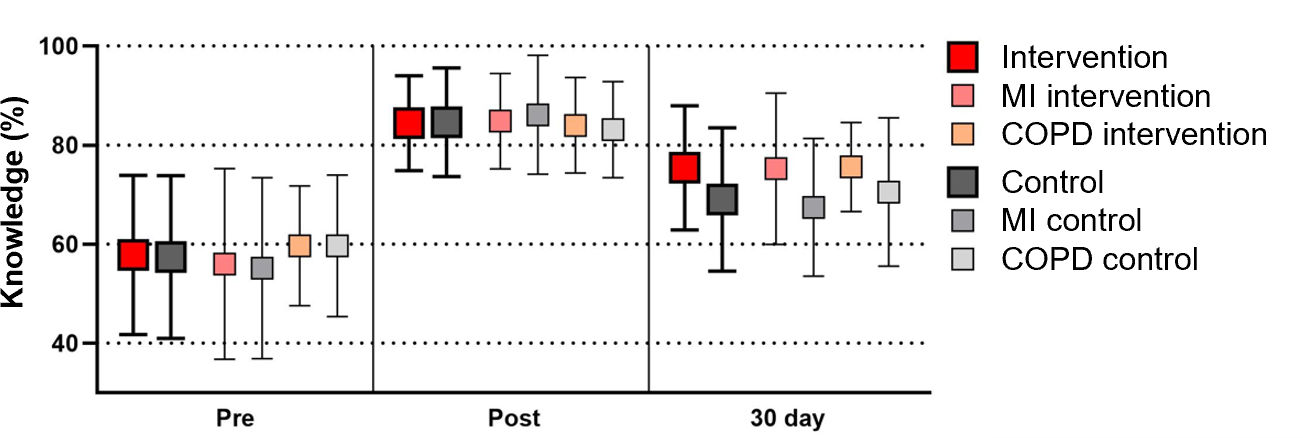
Knowledge test results as percentages of correct responses before (pre), immediately after (post), and 30 days after the intervention. Data are given in mean (box) with corresponding error bars (SD). The larger symbols and lines indicate overall results, while the smaller symbols detail the results for the individual scenarios. COPD: chronic obstructive pulmonary disease; MI: myocardial infarction; VR: virtual reality.

Interrater reliability was consistently high in the knowledge test with a Cohen κ of 0.927 averaged across all questions. Student performance is depicted in Figure 3. The previous knowledge of participants was similar in both the intervention and control condition for the pretest (intervention: mean 57.8%, SD 16.1%; control: mean 57.4%, SD 16.4%; difference: −0.4, 95% CI −5.8 to 4.9). Both conditions exhibited a similar short-term increase immediately following the respective learning sessions (posttest intervention: mean 84.4%, SD 9.6%; control: mean 84.6%, SD 11%; difference: 0.2, 95% CI −3.2 to 3.6). However, the 30-day retention test revealed differences; knowledge acquired in the VR scenario was retained significantly better than that acquired from the video seminar (intervention: mean 75.4%, SD 12.5%; control: mean 69.0%, SD 14.5%; difference: −6.4, 95% CI −10.9 to −1.9). At the level of scenarios, the difference in the 30-day retention test was more noticeable for the case of myocardial infarction (intervention: mean 75.2%, SD 15.3%; control: mean 67.4%, SD 13.9%; difference: −7.8, 95% CI −14.8 to −0.8). Conversely, for chronic obstructive pulmonary disease, the difference was slightly less pronounced (intervention: mean 75.6%, SD 9%; control: mean 70.5%, SD 15.3%; difference: −5.1, 95% CI −11.0 to 0.9). To account for previous knowledge, short-term gains (intervention: mean 26.6%, SD 15.3%; control: mean 27.2%, SD 16%; difference: 0.6, 95% CI −4.5 to 5.8) and long-term gains (intervention: mean 17.8%, SD 15.1%; control: mean 11.9%, SD 18%; difference: −5.9, 95% CI −11.5 to −0.4) were calculated.

On self-assessment by participants, the 2 items of estimated learning success in the VR scenarios approached the maximum possible value of 5 (didactic value: mean 4.83, SD 0.41; individual learning benefit: mean 4.85, SD 0.40). In contrast, these items in the control condition had lower scores, only slightly above average (didactic value: mean 3.44, SD 1.00; individual learning benefit: mean 3.15, SD 1.15). Furthermore, the differences between the conditions were statistically significant (each *P*<.001).

### Physiological Stress Markers and Perceived Stress During VR Scenarios

**Figure 4.**
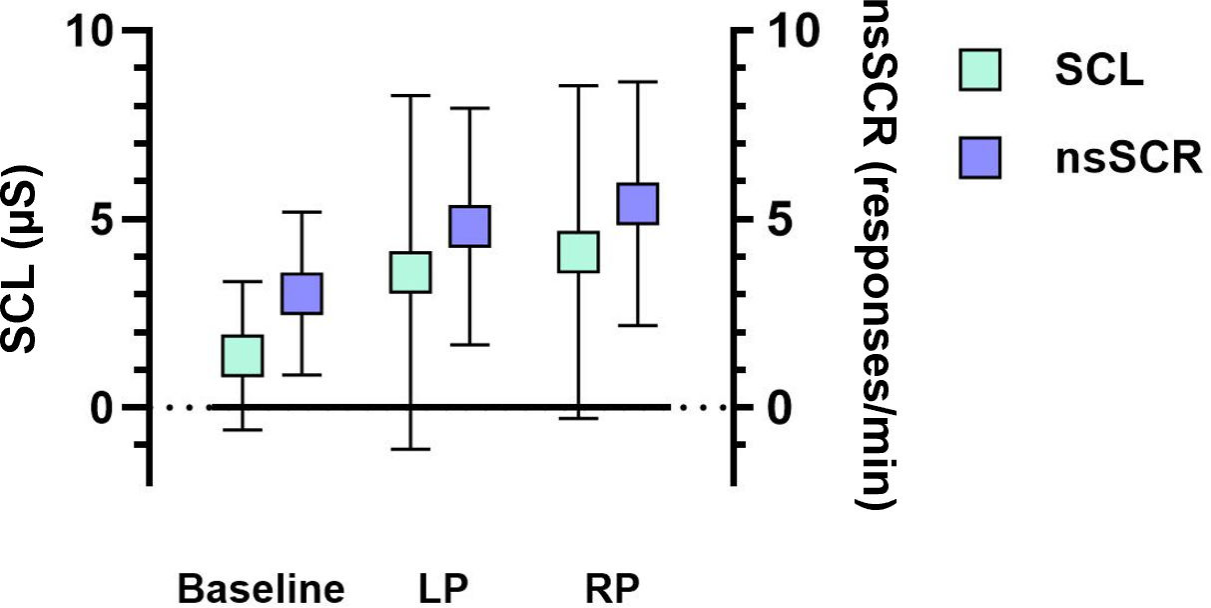
The progression of physiological stress markers SCL (µs) and nsSCR (responses/minute) during the completion of the virtual reality scenarios. Data are given in mean (box) with corresponding error bars (SD). LP: learning phase; nsSCR: nonspecific skin conductance response; RP: repetition phase; SCL: skin conductance level.

For all participants, parameters of EDA were recorded during the completion of the VR scenarios, as illustrated in Figure 4. SCL and nsSCR exhibited increases from baseline values (mean 1.39, SD 1.98 µs and mean 3.05, SD 2.16 per min, respectively) to higher levels during the LP (mean 3.63, SD 4.71 µs and mean 4.81, SD 3.16 per min, respectively), further rising to their peak values during the RP (mean 4.16, SD 4.43 µs and mean 5.38, SD 3.26 per min, respectively). The differences between the average values of LP and RP for both SCL and nsSCR and their respective baseline values were highly significant (both *P*<.001). Throughout the study, there was considerable variability in the values, as indicated by relatively high SDs.

Within the perceived stress questionnaire used in the VR scenarios, the factor “challenge to perform” received average ratings (mean 3.00, SD 1.08) among participants, followed by the “sense of control” (mean 2.51, SD 0.95) and low ratings for “social interaction to learn” (mean 1.37, SD 0.80). Accordingly, moderate levels were reported for the “total perceived stress” item (mean 2.86, SD 0.94).

### Analysis of Correlations

**Figure 5.**
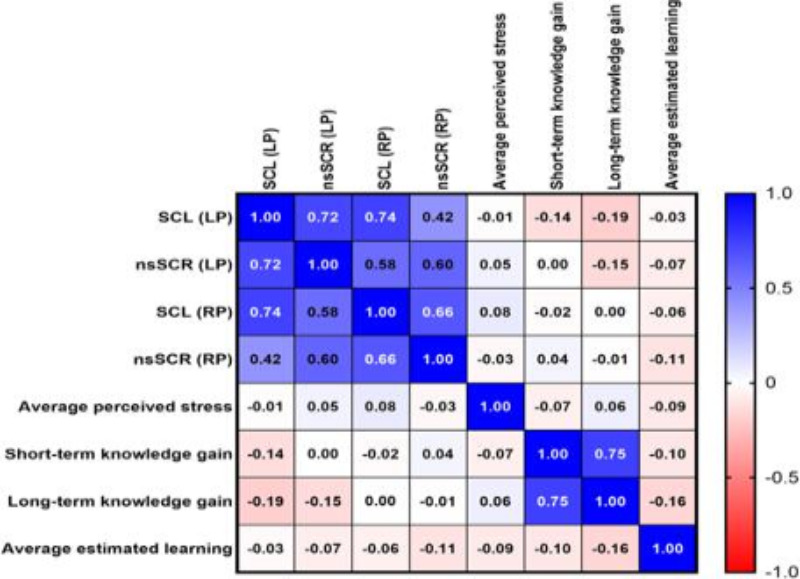
Correlation matrix between stress markers, knowledge gains, average perceived stress, and average estimated learning. The heatmap scale (right) indicates the color coding for positive (blue) and negative (red) correlations. The more intense the color, the higher the correlation coefficient. Corresponding significance values are listed in Multimedia Appendix 4. LP: learning phase; nsSCR: nonspecific skin conductance response; RP: repetition phase, SCL: skin conductance level.

To investigate any potential influence of the baseline-corrected physiological stress markers (SCL and nsSCR) on short or long-term knowledge gains and students’ self-assessments (average perceived stress and average learning gain), correlations were calculated (Figure 5). The parameters within the same modality (knowledge gains and stress markers) correlated well with each other. However, weak and nonsignificant negative correlations were observed between short- and long-term knowledge gains and baseline-corrected physiological stress markers recorded during the LP. The respective correlations were even less pronounced during the RP. Subsequently, quadratic regression analyses were performed to explore further the relationships between knowledge gains and average perceived stress as well as baseline-corrected physiological stress markers, respectively. While some plots visually suggested the presence of inverted U-shaped trendlines, all *R*^2^ values fell below 0.2, indicating only weak correlation (data not illustrated).

Average perceived stress and average estimated learning exhibited only minimal correlation with knowledge gains and baseline-corrected physiological stress markers (all ρ<0.2). Notably, there was no correlation between average perceived stress and average estimated learning (ρ=–0.09). All ρ values are depicted in Figure 5 and corresponding *P* values are listed in Multimedia Appendix 4.

To evaluate the potential influence of (nominally and ordinally distributed) person-specific factors, group comparisons were conducted. No significant differences were found between gender, academic semester, previous experience level in emergency medicine, or experience with 3D or VR applications in relation to short- and long-term knowledge gain or baseline-corrected physiological stress markers.

## Discussion

### Principal Findings

This study, conducted within the curricular framework of a degree course in medicine, used a randomized, controlled design to assess short- and long-term knowledge gains, as well as estimated learning success resulting from self-moderated learning in a complex VR-based medical emergency training. Furthermore, we investigated the influence of physiological stress markers and perceived stress among students.

Focusing on our primary objective, we found significantly greater knowledge retention after 30 days following a 35-minute self-moderated VR learning session with automated feedback, when compared with a video seminar of equivalent duration and content. The moderate effect size must also be considered in the context of the high previous knowledge (almost 60%) and the very explicit presentation of medical facts in the control condition. While VR-based programs are increasingly integrated into medical curricula, small sample sizes and heterogeneous study designs hinder clear conclusions on the effectiveness of learning with such programs [32]. To date, the number of randomized, controlled trials is limited, and results are partly contradictory. For VR-based resuscitation training, 2 studies reported different results. The VR-training group had either better or worse no-flow times, dependent on which control group was chosen (web-based versus “classic” basic life support course) [4,33]. Another study examining the effectiveness of VR-based pediatric intubation training reported similar scores compared with learning with a checklist [34]. Although it is known that context-specific knowledge tends to be anchored in memory better, we found no studies that included long-term outcomes by measuring knowledge or skill retention in VR-based emergency training. However, only training methods that demonstrate long-term effects are likely to help close the knowledge and skill gaps observed in newly graduated physicians. By allowing learners to engage with highly immersive case scenarios—complete with automated feedback and the option for repetitive, self-directed practice—VR-based training addresses these deficits in ways that traditional methods alone often cannot [10,35]. Such simulations allow repeated practice of high-stakes actions and decision-making without endangering patients, which is especially relevant for complex or infrequent emergencies where hands-on exposure in real clinical settings can be hard to achieve [36]. Furthermore, time constraints in medical curricula frequently limit comprehensive hands-on training. In this regard, self-moderated VR sessions offer a resource-efficient alternative; learners can work independently, and tutors or faculty need only intervene selectively. This flexibility may reduce the strain on educators and infrastructure, making it feasible to revisit core competencies regularly in the future.

We could not observe a relevant correlation between knowledge gains and estimated learning success in this study. Even though subjective learning outcomes are frequently used in educational research [37,38] (including our own work [13]), a discrepancy between subjective and objective measures has been observed in various medical disciplines. Both medical students and junior doctors often face challenges in accurately self-assessing their own competence in various clinical skills [14,15]. Research from cognitive science suggests that self-assessment of learning is influenced more by affective factors, such as enthusiasm for a subject or a teaching method, rather than by objective knowledge gain [39]. This seems particularly plausible in the context of engaging and innovative teaching methods that predominantly elicit positive emotional responses among learners. We conclude that self-reported learning outcomes—especially those from such methods—should ideally be supplemented or even substituted by objective assessments of competence.

The second objective of our study was to quantify physiological stress responses during VR-based learning and correlate them with levels of perceived stress. We successfully measured 2 physiological stress markers, SCL and nsSCR, in all participants. Both parameters exhibited similar increases during the scenarios, consistent with literature benchmarks [23]. Current evidence suggests that autonomic nervous system responses may be less pronounced in VR settings compared with real-life situations [40], yet more prominent than those observed in similar 2D screen settings [41]. Notably, SCLs were higher than average in stress-inducing VR-based disaster scenarios [42] and from VR environments designed specifically to trigger stress [43], indicating an authentic representation of the VR case scenarios. It is surprising that both physiological stress markers were lower during the LP (presumably the more stressful activity) than during the RP (where learning content was supposed to be reproduced). This is plausible for SCL, which can drift toward higher values over the recording period under certain circumstances (eg, change in skin temperature), as reported [23]. However, we also observed a similar increase in nsSCR during the RP, indicating that the rise from LP to RP was not merely an artifact of SCL. One possible explanation is that participants might have found it challenging to concentrate on reproducing the medical procedures during the RP, especially after just receiving (potentially poor) results from the final evaluation. This type of stress during the RP might differ qualitatively from that experienced during the LP, potentially explaining the differing correlation levels observed between the 2 phases (described further in this study). Interestingly, we found no correlation between the physiological stress markers and the identified items for perceived stress and the average perceived stress, respectively. This finding contrasts with a recent review, which reported that the majority (approximately 87%) of studies across various contexts found a positive association between EDA and perceived stress [44]. However, the reported degree of correlation varies strongly even within the specific context of simulation training, from no correlation in a simulated airway emergency [45] to a very high correlation during surgical skills training [46]. These findings underscore the need for further research into different measures of stress responses in VR settings (eg, heart rate variability or salivary cortisol) to improve our understanding and contextualize our results.

Finally, we explored the relationship between physiological stress markers and perceived stress in relation to knowledge outcomes. Our findings revealed a weak negative correlation between physiological stress markers recorded during the LP and long-term knowledge gain, while correlations with stress markers recorded during the RP were largely absent. It is generally recognized that affects and emotions play a pivotal role in influencing learning and memory performance [28]. Nonetheless, the exact relationship between physiological stress parameters and learning or performance outcomes remains somewhat ambiguous. For instance, in similar training sessions designed to establish intraosseous access [47], conduct pediatric intubation [48], or execute simulated assessments [49], key physiological stress markers like salivary cortisol and heart rate variability displayed no correlation with performance outcomes. Contrastingly, broader data from simulation contexts generally suggest that stress adversely affects learning outcomes [20,50] and that stress management training can improve performance during critical simulated situations [30]. The lack of correlation between average perceived stress and average estimated learning success aligns with the divergent objective outcomes (knowledge and physiological stress response). Mathematically, this was expected given that subjective and objective measures within the same domain (knowledge or stress) did not correlate in our study. The reason we were unable to reproduce the correlations between the perceived stress and estimated learning success found in our previous study [13] may lie, in the simplified questionnaire structure, which replaced all items of a factor (eg, sense of control) with a single question. To elucidate the discrepancies among subjective and objective measures of stress and their relation to knowledge outcomes further, a future research direction could involve a more detailed qualitative approach, such as the repeated assessment of subjective stress dimensions throughout the VR scenario. This method could provide deeper insight into how stress dynamics uniquely affect individual learning trajectories in immersive virtual environments.

### Strengths

The study benefited primarily from a relatively large sample size, which was representative of the medical student population at the study location. Knowledge was assessed at multiple time points and results largely corresponded across 2 scenarios with distinct content, thereby increasing the generalizability of the study. Established physiological stress markers, such as SCL and nsSCR, were recorded using validated tools alongside a baseline measurement, augmenting the robustness of the findings. The greatest strength, however, is the randomized, controlled, and single-blinded study design in which these measures were conducted.

### Limitations

Several factors limit the generalizability of this study, however. First, the sample consisted of medical students from a single academic institution, which may not reflect broader demographics. Second, the learning sessions for participants were relatively short, with a duration of only 35 minutes. It would be interesting to explore the (dose-dependent) effects of longer or repeated learning sessions. Third, the participants in this study were not blinded regarding whether they were currently receiving the intervention or control condition, which may have influenced their performance. We aimed to minimize at least the motivational bias by using an intraindividual cross-over design, where each participant received both the intervention and control in different case scenarios. Finally, our study focused solely on applied knowledge. Given the simplified representation of invasive procedures in the program and the lack of haptic feedback from VR controllers, we chose not to assess practical skills. Future research should investigate whether VR can effectively support long-term skill-based learning outcomes.

### Conclusion

This study provides new insights into the durability and effectiveness of learning outcomes achievable through VR technology in emergency simulation training implemented in a medical degree program. The results suggest that the immersive, practical learning experiences offered by VR-based scenarios may yield long-term educational benefits, although research on longer retention periods is still lacking. Given the time constraints faced by clinical educators and the heterogeneity of curricula regarding complex emergencies, self-moderated VR-based learning emerges as a valuable complement to medical education.

While real-time measurement of physiological stress markers in VR scenarios would be desirable to improve the learning experience (eg, by dynamically adjusting the level of difficulty based on stress data), this study could not establish a correlation between measured stress and learning outcomes. More sophisticated methods may be required to clarify this relationship.

## Supplementary material

10.2196/67412 Multimedia Appendix 1 Feedback components in the adapted software (video).

10.2196/67412 Multimedia Appendix 2 Rubric for open-ended questions (German and English) applied for knowledge tests.

10.2196/67412 Multimedia Appendix 3 Subjective measures questionnaire containing questions on perceived stress and estimated learning success (1 to 5 on a 5-point Likert scale) as well as questions on demographic information.

10.2196/67412 Multimedia Appendix 4 *P* values of the correlation matrix (Figure 3) between baseline-corrected parameters of the physiological stress response and knowledge tests at different time points, as well as the subjective measures of perceived stress and estimated learning success. *P* values below the significance level of .05 are printed in bold. LP: learning phase; RP: repetition phase; SCL: skin conductance level; nsSCR: nonspecific skin conductance response.

10.2196/67412 Checklist 1 CONSORT-EHEALTH (Consolidated Standards of Reporting Trials of Electronic and Mobile Health Applications and Online Telehealth) checklist.

## Abbreviations

CONSORT-EHEALTH: Consolidated Standards of Reporting Trials of Electronic and Mobile Health Applications and Online Telehealth
EDA: electrodermal activity
LP: learning phase
nsSCR: nonspecific skin conductance response
RP: repetition phase
SCL: skin conductance level
STEP-VR: Simulation-based Training of Medical Emergencies for Physicians Using Virtual Reality
VR: virtual reality
