# Supplementary material for: Knowledge Gain and the Impact of Stress in a Fully Immersive Virtual Reality–Based Medical Emergencies Training With Automated Feedback: Randomized Controlled Trial
Source: J Med Internet Res. 2025 Jun 4;27:e67412. doi: 10.2196/67412 (PMC12154946; doi:10.2196/67412)
Supplement: Multimedia Appendix 2 [file jmir-v27-e67412-s002.docx]

# Bewertungsmaßstab für Wissenstests (German version)

# Szenario A Myokardinfarkt

1. Nach welcher Zeit sollte bei einem möglichen akuten Koronarsyndrom spätestens ein 12-Kanal-EKG geschrieben worden sein? Angabe bitte in Minuten. (1P)
   - Die maximal zulässige Verzögerung beträgt hier 10 Minuten. (1P bei Wert +/- 10% Abweichung
     *(Leitlinie „Therapie des akuten Herzinfarkts bei ST-Streckenhebung (STEMI)“, 2017)*
2. Was ist die vordringlichste therapeutische Maßnahme beim ST-Hebungsinfarkt? Nennen Sie diese. (1P)
   - Herzkatheteruntersuchung; Perkutane koronare Intervention (PCI); Aktivierung des Herzkatheterrufdienstes (1P)
     *(Leitlinie „Therapie des akuten Herzinfarkts bei ST-Streckenhebung (STEMI)“, 2017)*
3. Sie haben den Verdacht auf ein akutes Koronarsyndrom. Welche Serummarker geben Ihnen nach Blutentnahme Aufschluss? Nennen Sie die zwei klinisch relevantesten. (2P)
   - kardiales Troponin *(wichtigster Marker!; Troponin T u. I sind Strukturproteine des kontraktilen Apparates der Herzmuskelzellen.)*, CK/CK-MB *(korreliert mit der Infarktgröße; organspezifische Isoform des Enzyms CK, die im Zytosol der Herzmuskelzellen auftritt u. als selektiver Marker für eine Myokardschädigung dient.)*, (je 1P bis max. 2P)
     *(Amboss: „Akutes Koronarsyndrom“, abgerufen 12/2021)*
4. Welche therapeutischen Maßnahmen sind bei kreislaufwirksamen Bradyarrhythmien (z.B. bei höhergradigem AV-Block) akut sinnvoll? (mehrere Antworten möglich) (3P)
   - Gabe von Atropin, Adrenalin, Orciprenalin (max. 2P); Anbringen eines temporären Schrittmachers (extern oder transjugulär) (1P)
     *(UpToDate „Third degree atrioventricular block“, abgerufen 02/2021)
     (Leitlinie „Therapie des akuten Herzinfarkts bei ST-Streckenhebung (STEMI)“, 2017)*
5. Welche beiden therapeutisch wirksamen Medikamente können bereits bei hochgradigem Verdacht auf ein akutes Koronarsyndrom verabreicht werden? Nennen Sie diese, falls möglich mit Dosisangabe. (2P)
   - Acetylsalicylsäure (ASS) 75-500mg i.v., unfraktioniertes Heparin 4000-10000IE oder 70IE/kgKG (alternativ niedermolekulares Heparin) (1P für Medikamentennamen + 1P für korrekten Dosisbereich)
     *(Leitlinie „Therapie des akuten Herzinfarkts bei ST-Streckenhebung (STEMI)“, 2017)*
6. Welche Begleitmedikamente sollten symptomorientiert verabreicht werden? Nennen Sie zwei Medikamente oder Wirkstoffe. (2P)
   - Opioidanalgetika (Morphin), Antiemetika (Dimenhydrinat), Nitrate (Glyceroltrinitrat), Anxiolytika (Benzodiazepin), Sauerstoffgabe (kein Medikament?!) (je 1P)
     *(Leitlinie „Therapie des akuten Herzinfarkts bei ST-Streckenhebung (STEMI)“, 2017)*
7. Wann ist beim akuten ST-Hebungsinfarkt (STEMI) die Gabe von Sauerstoff indiziert? Nennen Sie die Bedingung. (1P)
   - Hypoxämie, O_2_-Sättigung < 90 %; PaO_2_ < 60 mmHg (1P)
     *(Leitlinie „Therapie des akuten Herzinfarkts bei ST-Streckenhebung (STEMI)“, 2017)*
8. Welches antithrombotische Medikament bzw. welche Medikamentenklasse ist zusätzlich zu den beiden Standards bei gesicherter Diagnose eines Myokardinfarkts indiziert? Nennen Sie dieses. (1P)
   - zweiter Thrombozytenaggregationshemmer (Ticagrelor, Prasugrel, Clopidogrel 🡪 Wirkstoffe allein auch gelten lassen?); P2Y_12_-ADP-Rezeptor-Inhibitor (1P)
     *(Leitlinie „Therapie des akuten Herzinfarkts bei ST-Streckenhebung (STEMI)“, 2017)*

Szenario B COPD

1. Wie kommt es am häufigsten zu akuten Exazerbationen einer COPD? Nennen Sie den Auslöser. (1P)
   - ca. 80% eindeutig infektbedingt *(z.B. Haemophilus influenzae o. Streptococcus pneumoniae)* (1P)
     *(Amboss: „Chronisch obstruktive Lungenerkrankung“, abgerufen 12/2021)*
2. Welche Diagnostik muss zur Beurteilung des Ausmaßes der Hyperkapnie durchgeführt werden und sollte zur Verlaufsbeurteilung ggf. wiederholt werden? Nennen Sie diese. (1P)
   - arterielle Blutgasanalyse (1P)
     *(GOLD Report: “Evidence-based strategy document for COPD diagnosis, management, and prevention”, 2020)*
3. Was sind kausal wirksame medikamentöse Maßnahmen bei der schweren infektexazerbierten COPD? Nennen Sie drei Maßnahmen, wenn möglich mit Wirkstoffbeispiel. (3P)
   - β2-Agonisten: SABA (Salbutamol, Fenoterol); Muskarinrezeptor-Antagonisten (Anticholinergika): SAMA (Ipratropiumbromid); Systemische Steroide (Prednisolon-Äquivalent); Antibiotika (Amoxicillin/Clavulansäure); Beruhigung/Anxiolyse (Morphin) (kausal wirksam?!) (je 1P bis max. 3P; Nennung von Wirkgruppe oder Wirkstoffbeispiel ausreichend)
     *(S2k-Leitline „Diagnostik und Therapie von Patienten mit chronisch obstruktiver Bronchitis und Lungenemphysem“, 2018)
     (Amboss: „Chronisch obstruktive Lungenerkrankung“, abgerufen 12/2021)*
4. Welche beiden Parameter sind neben dem O_2_-Partialdruck in der Blutgasanalyse noch von entscheidender Bedeutung? Nennen Sie diese. (2P)
   - pCO_2_, pH-Wert (je 1P) (nur die beiden oder auch Parameter wie BE?)
     *(GOLD Report: “Evidence-based strategy document for COPD diagnosis, management, and prevention”, 2020)*
5. Welche weitere Labor-Diagnostik (inkl. infektiologischer Diagnostik) sollte beim V.a. infektexazerbierte COPD vordringlich erfolgen? Nennen Sie zwei Maßnahmen. (2P)
   - Blutentnahme mit Infektparametern *(Leukozyten, CRP, ggf. Procalcitonin)*; Abnahme von Blutkulturen; Sputumdiagnostik; Pneumokokken/Legionellen Antigen-Nachweis im Urin (je 1P bis max. 2P)
     *(S2k-Leitline „Diagnostik und Therapie von Patienten mit chronisch obstruktiver Bronchitis und Lungenemphysem“, 2018)*
6. Welche weitere apparative Diagnostik sollte erfolgen? Nennen Sie zwei Maßnahmen. (2P)
   - EKG, konventionelles Röntgen-Thorax, ggf. Ultraschall (je 1P bis max. 2P)
     *(S2k-Leitline „Diagnostik und Therapie von Patienten mit chronisch obstruktiver Bronchitis und Lungenemphysem“, 2018)*
7. Wann sollte der Wechsel von einer O_2_-Gabe zur nichtinvasiven Beatmung (NIV) erfolgen? Nennen Sie die Indikation für eine NIV-Therapie im Rahmen einer akuten exazerbierten COPD. (1P)
   - hyperkapnisches/ventilatorisches Versagen; akute hyperkapnische respiratorische Insuffizienz; pO2↓ u. pCO2↑; respiratorischer Azidose; pH↓; pCO2 > 65 mmHg (1P)
     *(S2k-Leitline „Diagnostik und Therapie von Patienten mit chronisch obstruktiver Bronchitis und Lungenemphysem“, 2018)*
8. Wie erfolgt die medikamentöse symptomatische Therapie der Dyspnoe bei einer schweren COPD-Exazerbation? Nennen Sie das Medikament oder die Wirkstoffklasse. (1P)
   - Morphin, bzw. Opioidanalgetika
     *(S2k-Leitline „Diagnostik und Therapie von Patienten mit chronisch obstruktiver Bronchitis und Lungenemphysem“, 2018)*

# Rubric for knowledge tests (English version)

# Scenario A: Myocardial Infarction

1. Within what time frame (in minutes) should a 12-lead ECG be performed in a suspected acute coronary syndrome? (1 point)
   - the maximum permissible delay is 10 minutes. (1 point for values within ±10%)
     *(ESC Guidelines for the management of acute myocardial infarction in patients presenting with ST-segment elevation, European Heart Journal; 2018 - doi/10.1093/eurheartj/ehx393)*
2. What is the primary therapeutic measure for ST-elevation myocardial infarction (STEMI)? Name the intervention. (1 point)
   - cardiac catheterization; percutaneous coronary intervention (PCI); activation of the catheterization laboratory team. (1 point)
     *(ESC Guidelines for the management of acute myocardial infarction in patients presenting with ST-segment elevation, European Heart Journal; 2018 - doi/10.1093/eurheartj/ehx393)*
3. You suspect an acute coronary syndrome. Which serum markers provide diagnostic confirmation after blood sampling? Name the two most clinically relevant markers. (2 points)
   - cardiac troponin (most important marker; Troponin T and I), CK/CK-MB (correlates with infarct size). (1 point per correct marker, max. 2 points)
     *(AMBOSS knowledge database: "Acute Coronary Syndrome", accessed 12/2021 on www.amboss.com)*
4. Which acute therapeutic measures are appropriate for hemodynamically relevant bradyarrhythmias (e.g., high-degree AV block)? Multiple answers possible. (3 points)
   - administration of atropine, adrenaline, or orciprenaline (max. 2 points); placement of a temporary pacemaker (external or transjugular) (1 point).
     *(UpToDate „Third-degree Atrioventricular Block“, accessed 02/2021 on www.uptodate.com)*
5. Which two therapeutically effective medications can be administered early in the presence of a high suspicion of acute coronary syndrome? Provide dosage if possible. (2 points)
   - acetylsalicylic acid 75–500 mg IV, unfractionated heparin 4000–10,000 IU or 70 IU/kg body weight (alternatively, low-molecular-weight heparin). (1 point for naming the medication, 1 point for providing the correct dosage range)
     *(ESC Guidelines for the management of acute myocardial infarction in patients presenting with ST-segment elevation, European Heart Journal; 2018 - doi/10.1093/eurheartj/ehx393)*
6. Which adjunctive medications should be administered based on symptomatology? Name two medications or active substances. (2 points)
   - opioid analgesics (e.g. morphine), antiemetics (e.g. dimenhydrinate), nitrates (e.g. glyceryl trinitrate), anxiolytics (e.g. benzodiazepines), oxygen therapy. (1 point per correct mention, max. 2 points)
     *(ESC Guidelines for the management of acute myocardial infarction in patients presenting with ST-segment elevation, European Heart Journal; 2018 - doi/10.1093/eurheartj/ehx393)*
7. When is oxygen therapy indicated in acute ST-elevation myocardial infarction (STEMI)? Name the criterion. (1 point)
   - hypoxemia: O₂ saturation < 90%; PaO₂ < 60 mmHg. (1 point)
     *(ESC Guidelines for the management of acute myocardial infarction in patients presenting with ST-segment elevation, European Heart Journal; 2018 - doi/10.1093/eurheartj/ehx393)*
8. Which additional antithrombotic drug or drug class is indicated alongside the two standard medications for a confirmed myocardial infarction? Name it. (1 point)
   - a second platelet aggregation inhibitor (e.g. ticagrelor, prasugrel, clopidogrel); P2Y_12_-ADP receptor inhibitor. (1 point)
     *(ESC Guidelines for the management of acute myocardial infarction in patients presenting with ST-segment elevation, European Heart Journal; 2018 - doi/10.1093/eurheartj/ehx393)*

# Scenario B: Chronic Obstructive Pulmonary Disease (COPD)

1. What is the most common trigger of acute exacerbations in COPD? Name the cause. (1 point)
   - approximately 80% of exacerbations are clearly infection-related (e.g. Haemophilus influenzae or Streptococcus pneumoniae). (1 point)
     *(AMBOSS knowledge database: "Chronic Obstructive Pulmonary Disease", accessed 12/2021 on www.amboss.com)*
2. Which diagnostic test is essential for assessing the extent of hypercapnia and should be repeated for monitoring? (1 point)
   - arterial blood gas analysis (1 point).
     *(GOLD Report: “Evidence-based strategy document for COPD diagnosis, management, and prevention”, 2020)*
3. What are the causally effective pharmacological interventions for severe infection-related COPD exacerbation? Name three drug classes with agent examples if possible. (3 points)
   - β₂-agonists: SABA (e.g. salbutamol, fenoterol); muscarinic receptor antagonists (anticholinergics): SAMA (e.g. ipratropium bromide); systemic steroids (e.g. prednisolone equivalent); antibiotics (e.g. amoxicillin/clavulanic acid); sedation/anxiolysis (e.g. morphine) (each 1 point, max. 3 points; drug classes or examples are accepted). *(DGP-S2k Guideline: „Diagnostik und Therapie von Patienten mit chronisch obstruktiver Bronchitis und Lungenemphysem" (Diagnosis and Treatment of Patients with Chronic Obstructive Bronchitis and Pulmonary Emphysema), 2018)
     (AMBOSS knowledge database: "Chronic Obstructive Pulmonary Disease", accessed 12/2021 on www.amboss.com)*
4. Besides oxygen partial pressure, which two parameters are critical in arterial blood gas analysis? (2 points)
   - pCO₂ and pH value (1 point each).
     *(GOLD Report: “Evidence-based strategy document for COPD diagnosis, management, and prevention”, 2020)*
5. Which further laboratory diagnostics (including infection diagnostics) should be performed urgently in suspected infection-related COPD exacerbation? Name two. (2 points)
   - blood sampling for infection parameters (e.g. leukocytes, CRP, possibly procalcitonin); blood cultures; sputum diagnostics; pneumococcal/legionella antigen detection in urine (each 1 point, max. 2 points).
     *(DGP-S2k Guideline: „Diagnostik und Therapie von Patienten mit chronisch obstruktiver Bronchitis und Lungenemphysem" (Diagnosis and Treatment of Patients with Chronic Obstructive Bronchitis and Pulmonary Emphysema), 2018)*
6. Which additional instrument-based diagnostics should be performed? Name two. (2 points)
   - ECG, conventional chest X-ray, possibly ultrasound (each 1 point, max. 2 points).
     *(DGP-S2k Guideline: „Diagnostik und Therapie von Patienten mit chronisch obstruktiver Bronchitis und Lungenemphysem" (Diagnosis and Treatment of Patients with Chronic Obstructive Bronchitis and Pulmonary Emphysema), 2018)*
7. When should oxygen therapy be switched to non-invasive ventilation (NIV)? State the indication for NIV therapy in acute exacerbated COPD. (1 point)
   - hypercapnic/ventilatory failure; acute hypercapnic respiratory insufficiency;
     pO2↓ and pCO2↑; respiratory acidosis; pH↓; pCO₂ > 65 mmHg. (1 point)
     *(DGP-S2k Guideline: „Diagnostik und Therapie von Patienten mit chronisch obstruktiver Bronchitis und Lungenemphysem" (Diagnosis and Treatment of Patients with Chronic Obstructive Bronchitis and Pulmonary Emphysema), 2018)*
8. How is the symptomatic treatment of dyspnoea in severe COPD exacerbation managed pharmacologically? Name the medication or drug class. (1 point)
   - morphine or opioid analgesics. (1 point)
     *(DGP-S2k Guideline: „Diagnostik und Therapie von Patienten mit chronisch obstruktiver Bronchitis und Lungenemphysem" (Diagnosis and Treatment of Patients with Chronic Obstructive Bronchitis and Pulmonary Emphysema), 2018)*
