# Supplementary material for: Knowledge Gain and the Impact of Stress in a Fully Immersive Virtual Reality–Based Medical Emergencies Training With Automated Feedback: Randomized Controlled Trial
Source: J Med Internet Res. 2025 Jun 4;27:e67412. doi: 10.2196/67412 (PMC12154946; doi:10.2196/67412)
Supplement: Multimedia Appendix 3 [file jmir-v27-e67412-s003.docx]

**Questionnaires on Perceived Stress, Estimated Learning Success and Demographic Information**

All used Likert scales had five steps with named endpoints 1 = fully disagree and 5 = fully agree.

1. **Questionnaire on Perceived Stress:**

Please respond to the questions based on the training method you just completed.

1.1 I felt stressed because many things in the scenario were beyond my control. (Likert scale)

1.2 I felt stressed because I lacked the expertise to handle the case. (Likert scale)

1.3 The presence of the tutor put me under pressure. (Likert scale)

1.4 Overall, I felt stressed while working through the case scenario. (Likert scale)

**2. Questionnaire on Estimated Learning Success:**

Please respond to the questions based on the training method you just completed.

2.1 The most recently completed training method was suitable as a learning tool for acquiring emergency medical knowledge. (Likert scale)

2.2 I personally benefited from the most recently completed training method in terms of my practical skills. (Likert scale)

**3. Questionnaire on demographic information and prior experience**

**If not otherwise mentioned, we used single-choice questions.**

3.1 Gender: diverse, male, female

3.2 Academic semester: 8th, 9th, 10th, >10

3.3 Career aspiration: Non-surgical specialty, Surgical specialty, Undecided

3.4 Do you have prior experience in internal medicine emergency care (e.g., clinical internship, prior healthcare training, voluntary service)? (yes/no)

3.5 If yes, what prior experience do you have in internal medicine emergency care? (free text)

3.6 How frequently do you play first-person action games (e.g., Minecraft, Counter-Strike) or narrative video games (e.g., Subnautica)? (five-point Likter scale with named endpoints 1 = never, 5 = often)

3.7 How much experience do you have with Virtual Reality simulations (total accumulated play/simulation time)? (no experience, <1 h, 1-5 h, 6-10 h, >10 h)

3.8 If you have prior experience with Virtual Reality simulations, in what context did you gain this experience? (free text)
