# Supplementary material for: Knowledge Gain and the Impact of Stress in a Fully Immersive Virtual Reality–Based Medical Emergencies Training With Automated Feedback: Randomized Controlled Trial
Source: J Med Internet Res. 2025 Jun 4;27:e67412. doi: 10.2196/67412 (PMC12154946; doi:10.2196/67412)
Supplement: Multimedia Appendix 4 [file jmir-v27-e67412-s004.docx]

Multimedia Appendix 1: P-values of the correlation matrix (Figure 3) between baseline-corrected parameters of the physiological stress response and knowledge tests at different time points, as well as the subjective measures of perceived stress and estimated learning success. P-values below the significance level of 0.05 are printed in bold.
LP: learning phase, RP: repetition phase, SCL: skin conductance level, nsSCR: non-specific skin conductance response

|  | **SCL (LP)** | **nsSCR (LP)** | **SCL (RP)** | **nsSCR (RP)** | **Average perceived stress** | **Short-term knowledge gain** | **Long-term knowledge gain** | **Estimated learning success** |
| --- | --- | --- | --- | --- | --- | --- | --- | --- |
| **SCL (LP)** |  | **<.001** | **<.001** | **<.001** | .91 | .26 | .12 | .83 |
| **nsSCR (LP)** | **<.001** |  | **<.001** | **<.001** | .67 | .99 | .23 | .56 |
| **SCL (RP)** | **<.001** | **<.001** |  | **<.001** | .52 | .89 | .00 | .60 |
| **nsSCR (RP)** | **<.001** | **<.001** | **<.001** |  | .78 | .73 | .94 | .36 |
| **Average perceived stress** | .91 | .67 | .52 | .78 |  | .58 | .59 | .43 |
| **Short-term knowledge gain** | .26 | .99 | .89 | .73 | .58 |  | **<.001** | .40 |
| **Long-term knowledge gain** | .12 | .23 | .00 | .94 | .59 | **<.001** |  | .18 |
| **Estimated learning success** | .83 | .56 | .60 | .36 | .43 | .40 | .18 |  |
